# Various Options for Scrotal Reconstruction: A Prospective Observational Study

**DOI:** 10.7759/cureus.22671

**Published:** 2022-02-27

**Authors:** Rajesh Maurya, Mohd Altaf Mir, Sumeet Mahajan

**Affiliations:** 1 Burns & Plastic Surgery, All India Institute of Medical Sciences, Bathinda, IND

**Keywords:** psychosexual, flap, grafting, gangrene, trauma, scrotum

## Abstract

Background

Scrotal defects in developing countries are common challenges for the reconstructive surgeon and hence this work has been done with the aim to compare the outcome, advantages and disadvantages of different modalities of scrotal reconstruction.

Methods

The prospective observational hospital-based study of reconstruction of scrotal defects following trauma and Fournier’s gangrene was done over a period of three years. Scrotal defect reconstruction was done in 35 patients by scrotal advancement flap, split thickness skin grafting, medial thigh flap, anterolateral thigh flap and groin flap keeping in mind the various indication of different modalities. The reconstructed scrotums were observed for flap survival and skin graft intake for seven to 10 days in the hospital and then were followed for three months in a follow-up clinic.

Results and observations

The mean age of our patients was 48.57±5.01 years. Most of the soft tissue defects of the scrotum were post-traumatic (83%). Scrotal reconstruction was done often by flaps and more frequently used flap for reconstruction of scrotum was scrotal advancement flap. All flaps and grafts survived well. Mean hospitalization time was highest for groin flap cover whereas mean operative time was highest for anterolateral thigh flap cover.

Conclusion

Every case of scrotal defect needs an individual approach for scrotal reconstruction depending upon patient age, general condition of the patient, wound status, and the patient’s requirement.

## Introduction

Trauma and Fournier’s gangrene can cause significant soft tissue loss involving the scrotum, penis and thighs. The soft tissue coverage of scrotal defects and exposed testes is vital for the protection of testicular function and sparing the sexual wellbeing of the person. Reconstruction is also important for perineal cosmesis and the psychological wellbeing of a patient. Various reconstructive procedures are split thickness skin grafting and flaps.

Trauma is the leading cause of soft tissue loss of the scrotum worldwide, however in developing countries Fournier’s gangrene is still contributing to a significant proportion of soft tissue loss of the scrotum.

Fournier’s gangrene is a rapidly progressive, fatal, necrotizing fasciitis involving skin and soft tissue of the scrotum and perineum. Several terms that have been used alternatively for Fournier’s gangrene are streptococcus gangrene, necrotizing fasciitis, periurethral phlegmon, phagedaena and synergistic necrotizing fasciitis [1].

Systemic conditions such as alcoholism, diabetes mellitus, paralysis or neurological deficit and immunosuppression are predisposing factors of Fournier’s gangrene [2]. Men are most commonly involved and it can present in any age group. Fournier’s gangrene may occur in children following insect bite and trauma [3].

It can cause significant soft tissue loss involving the scrotum, penis and thighs. Since the testes have an independent vascular supply, they survive but may have secondary function loss. After debridement and control of infection, skin coverage of scrotal defects and exposed testes is indispensable.

Reconstruction of the scrotum is important for functional, cosmetic and psychological wellbeing [4]. Various techniques that have been described for the reconstruction of these defects are split thickness skin grafts (STSG) [5], muscle flaps [6], fascio-cutaneous flaps [7], perineal flap [8], anterolateral thigh flap (ALT) [4], deep inferior epigastric perforator flap [9] and anteromedial thigh flap (AMT) [10].

Scrotal defects in developing countries are a common challenge to the reconstructive surgeon and hence this work has been done with the aim to compare the outcome, advantages and disadvantages of different modalities of scrotal reconstruction.

## Materials and methods

Study design

The prospective observational hospital-based study of reconstruction of scrotal defects following trauma and Fournier’s gangrene was done over a period of three years. The study was conducted at the first two author’s previous institution after institutional ethical approval and is registered vide reference number 642/FM/17.

Study population and sample size

**Figure 1:**
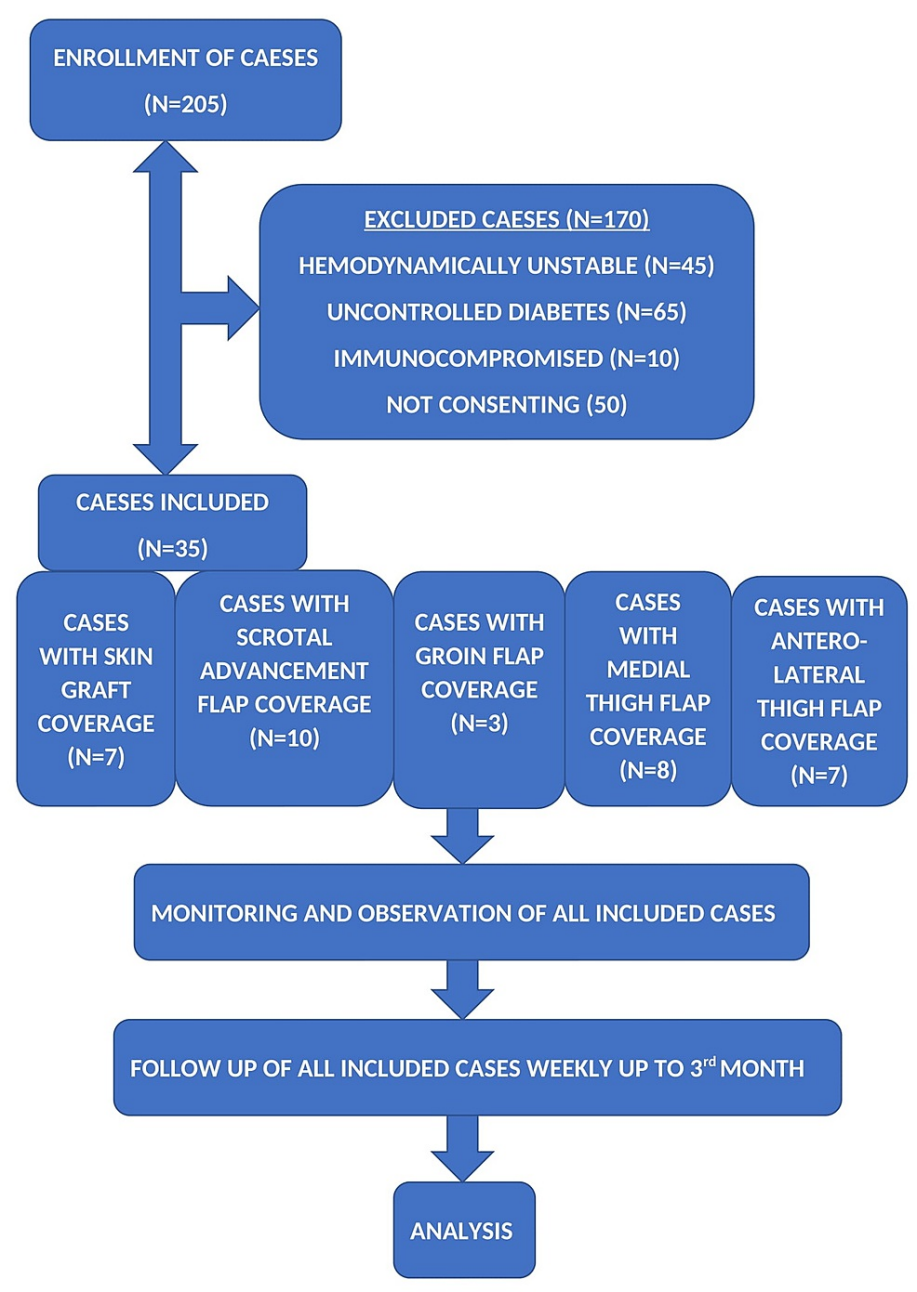
Study design Flow diagram depicting the number of cases recruited, excluded and included. It also show the number of cases in each mode of management and the overall study design from recruitment of cases to end of follow up.

Patients with soft tissue defects of the scrotum following trauma (n=29) and Fournier’s gangrene (n=6) who underwent reconstruction were included in the study and observed prospectively during the hospitalized period and then afterward for three months. The sample size calculation was performed using OpenEpi (OpenEpi, Version 3, open-source calculator-SS Cohort) online tool. Considering 80% power with 5% of significance and high effect size, the sample size calculated was 35. The study design and population are described in Figure 1.

Selection criteria for study participants

All patients from 16-59 years of age with scrotal soft tissue defects who underwent tissue coverage were included in the study. Children of less than 16 years and elderly of more than 59 years, hemodynamically unstable patients, immunocompromised patients, uncontrolled diabetes and critically ill patients not fit for anesthesia were excluded.

The scrotal defect coverage was done by various method chosen according to size of defect, age and comorbidities of the patient. In our study scrotal defect reconstruction was done in 35 patients with scrotal advancement flap, split thickness skin grafting, medial thigh flap , anterolateral thigh flap and groin flap, keeping in mind various indications for different modalities. Patients were observed for seven to 10 days in ward for skin graft take clinically and flap viability clinically and using hand held vascular doppler. After discharge from hospital the patients were followed weekly in a follow-up clinic for three months addressing size of scrotum, color and hair bearing skin of scrotum, scar appearance at scrotum and donor site morbidity.

The aesthetic outcome of operated patients was evaluated at two months of follow-up through Likert scale based on a questionnaire that included assessment of size of scrotum, shape and appearance, scrotum color and donor site scar by three independent plastic surgeons. The satisfaction score was calculated as per Table 1.

**Table 1:** Likert scale of satisfaction after scrotal reconstruction. The parameters for satisfaction score used for aesthetic assessment after reconstruction by three independent Plastic Surgeons.

Criteria	Very unsatisfied	Unsatisfied	Satisfied	Very satisfied
Size of scrotum	1	2	3	4
Shape and appearance of scrotum	1	2	3	4
Color	1	2	3	4
Donor site scar	1	2	3	4

Data collection and statistical analysis

The data was recorded as per set Performa which was then tabulated in a Microsoft Excel spread sheet. The data has been analyzed using Statistical Package for Social Sciences (SPSS) version 26.0 (IBM Corp., Armonk, NY, USA). The results are expressed as mean ± standard deviation (SD) for discrete data and proportions for descriptive data.

## Results

**Figure 2:**
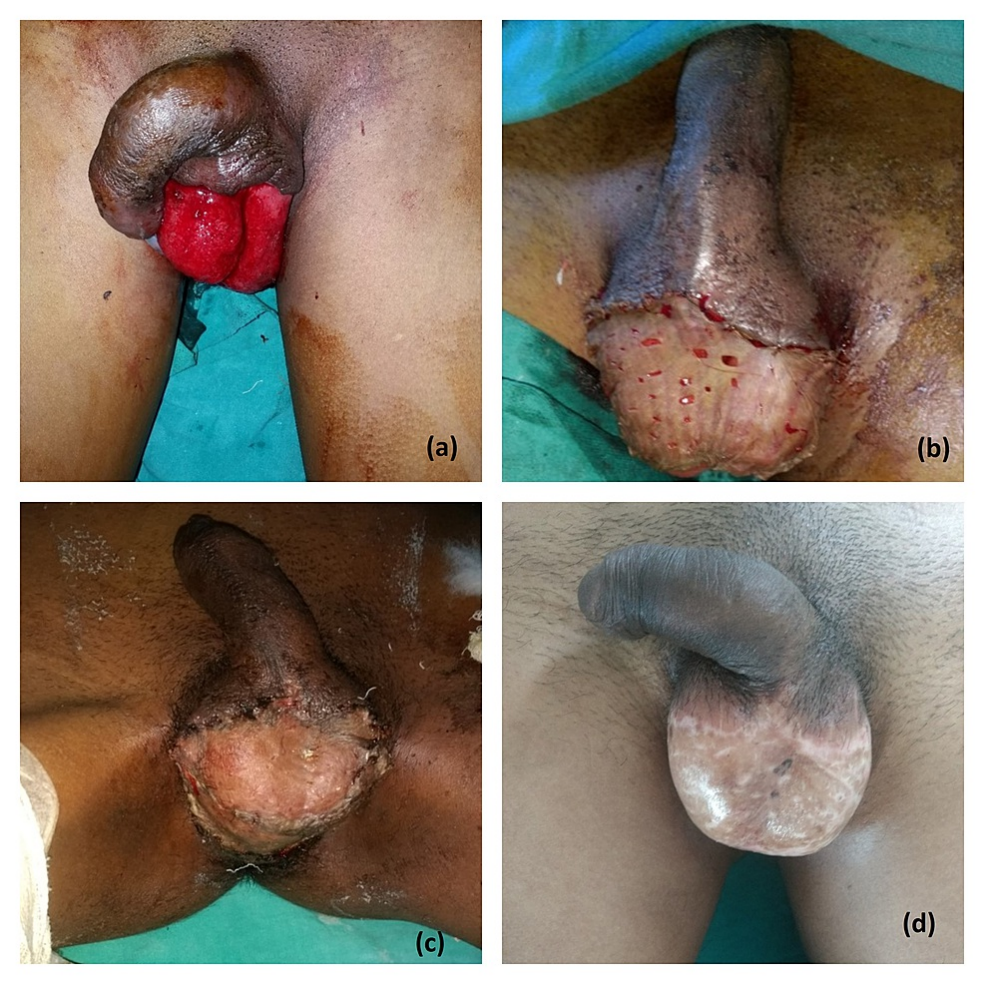
Split thickness skin grafting (STSG). Management of soft tissue defects of the scrotum with STSG. Panel a shows scrotal soft tissue defect, panel b shows STSG cover, panel c is a post-operative image suggesting satisfactory graft uptake and panel d depicts final results at three months follow-up.

**Figure 3:**
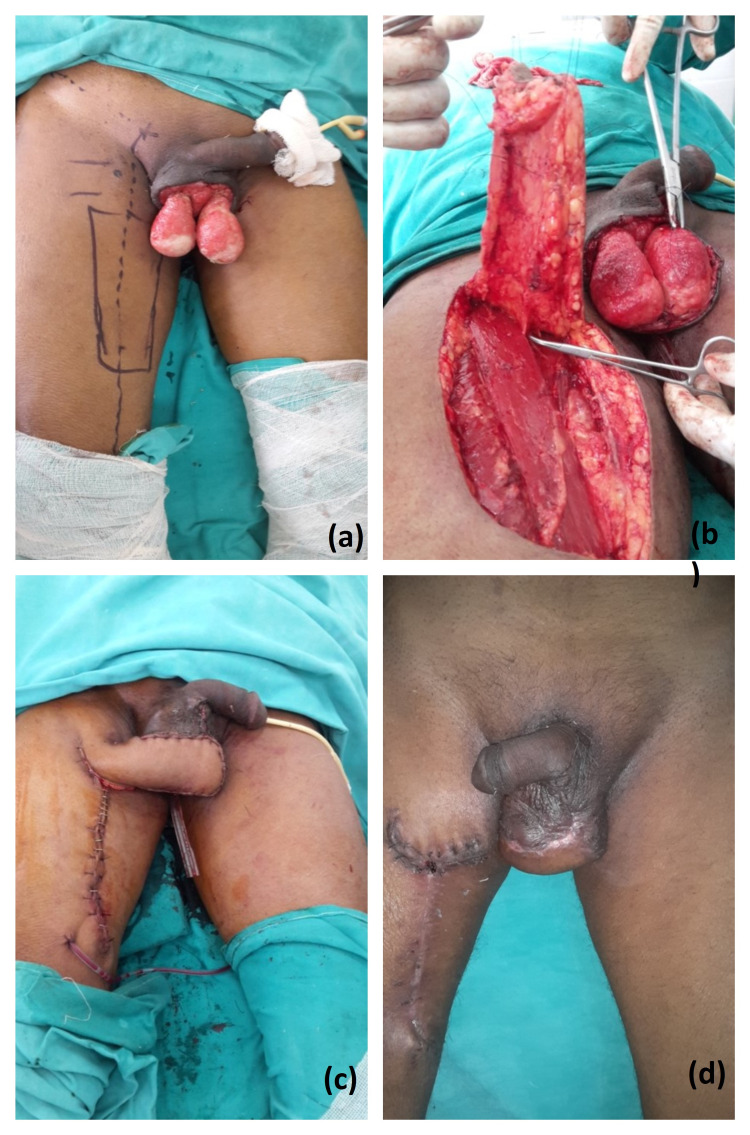
Medial thigh flap coverage. Panel a depicts a soft tissue scrotal defect and medial thigh flap marking, panel b shows raised medial thigh flap and supplying perforator, panel c shows immediate flap coverage of the scrotal defect and panel d is a follow-up photograph at two weeks suggesting healthy flap.

**Figure 4:**
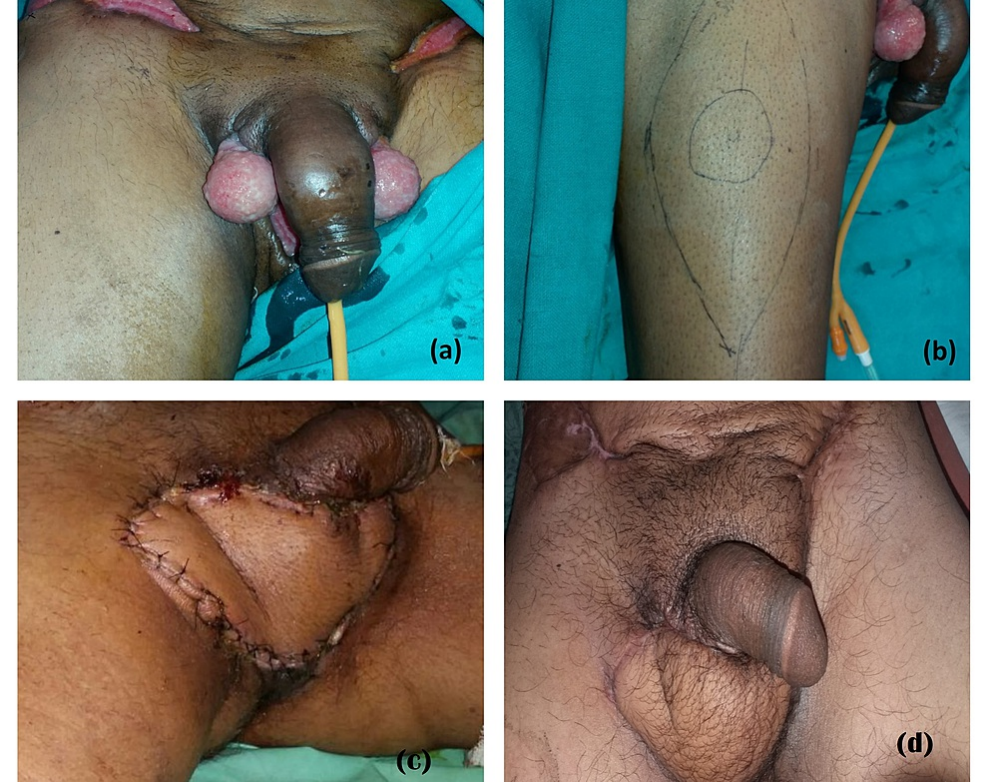
Anterolateral thigh (ALT) flap coverage. Panel a depicts a soft tissue scrotal defect with exposed testicles, panel b shows ALT flap marking, panel c shows flap coverage of the defect and panel d shows healed flap at three months follow-up.

**Figure 5:**
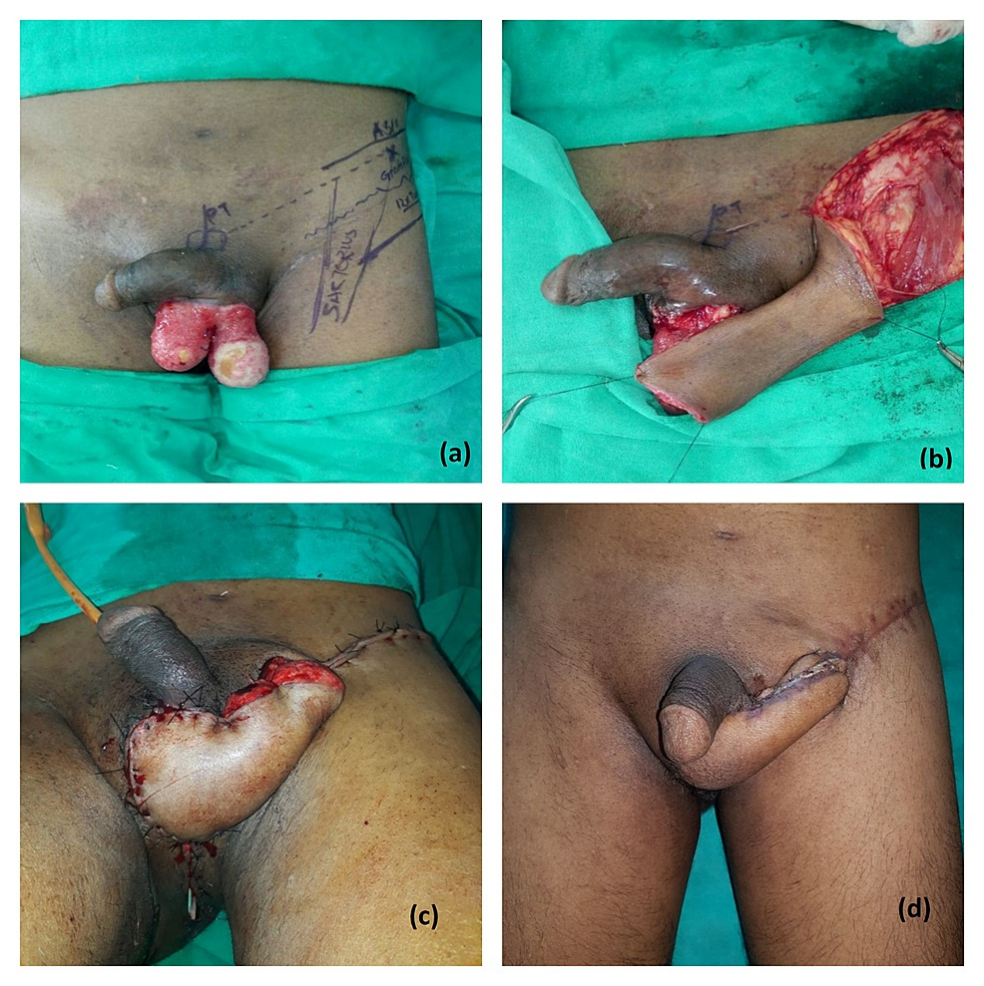
Groin flap coverage. Panel a shows soft tissue defect of scrotum with exposed testicles, panel b shows raised groin flap and arch of rotation of the flap, panel c depicts immediate flap coverage of the scrotal defect and panel d suggests healthy flap at two months follow-up.

The mean age of our patients was 48.57±5.01 years. Most of the soft tissue defects of the scrotum were post-traumatic (83%) and Fournier’s gangrene contributed to 17%. The soft tissue defects after Fournier’s gangrene often required wound optimization before tissue coverage. The scrotal defect coverage was done with various methods chosen according to size of defect and age and comorbidities of patient. In our study scrotal defect reconstruction was done in 35 patients by scrotal advancement flap, split thickness skin grafting (Figure 2), medial thigh flap (Figure 3), anterolateral thigh flap (Figure 4) and groin flap (Figure 5) keeping in mind various indications for different modalities.

The scrotal reconstruction was done often by flaps and the most frequently used flap for reconstruction of the scrotum was scrotal advancement flap. All flaps and grafts survived well. Mean hospitalization time was highest for groin flap cover whereas mean operative time was highest for anterolateral thigh flap cover. Various complications seen in patients treated with different reconstructive techniques are mentioned in Table 2. 

**Table 2:** Various reconstructive techniques and their outcomes. (n) -Number in brackets in complications indicates the number of patients.

Procedure	Total number of patients (n=35)	Mean Hospitalization time ± SD (in days)	Mean operative time ± SD (in minutes)	Complications
Scrotal advancement flap	10 (28.57%)	8.7 ± 2	50 ± 4.5	Nil
Split thickness skin grafting	7 (20%)	9.4 ± 2.3	60 ± 4.6	-Infection at recipient site (n=1)
				-Perineo-scrotal gap (n=1)
Medial thigh flap cover	8 (22.86%)	15.8	120 ± 29.9	-Partial distal necrosis of flap(n=2)
				-Un-recovered sensation (n=1)
				-Bulky flap (n=1)
				-Wound dehiscence (n=1)
				-Infection of suture line (n=1)
Anterolateral thigh flap cover	7 (20%)	16.1	150 ± 32.9	-Marginal flap necrosis (n=2)
				-Bulky flap (n=3)
				-Wound dehiscence and infection (n=2)
Groin flap cover	3 (8.57%)	18	105 ± 24.4	Nil

The aesthetic outcomes were evaluated through Likert scale based on a questionnaire that included assessment of the size of the scrotum, shape and appearance, scrotum color and donor site scar by three independent plastic surgeons. The aesthetic outcome was highly satisfactory after scrotal advancement flaps with mean ± SD score of 11.5 ± 1.61 followed by anterolateral thigh flap 11.4 ± 1.98 and least after skin grafting 9.7 ± 1.99. A summary of aesthetic results is given in Table 3.

**Table 3:** Summary of aesthetic results. The table shows aesthetic outcome was highly satisfactory after scrotal advancement flaps followed by anterolateral thigh flap and least after skin grafting.

Procedure	Number of patients	Mean Satisfaction score ± SD
Scrotal advancement flap	10	11.5 ± 1.61
Split thickness skin grafting	7	9.7 ± 1.99
Medial thigh flap cover	8	11.3 ± 2.01
Anterolateral thigh flap cover	7	11.7 ± 1.98
Groin flap cover	3	10.3 ± 1.87

After the completion of our study, we were able to derive the following advantages and disadvantages of various modalities of scrotal reconstruction, which are tabulated below in Table 4. Consideration of the listed advantages and disadvantages of these different flaps and STSG in Table 4 can help in decision making of the type of flap required in different cases of scrotal soft tissue defects in different clinical scenarios. Table 4 describes the advantages and disadvantages in detail.

**Table 4:** Advantages and disadvantages of different modalities of scrotal reconstruction. STSG: split thickness skin graft; ALT: anterolateral thigh flap

Name of procedure	Advantages	Disadvantages
Delayed primary closure	Follow the principle of replace like with like	For small defect only
	Reconstruction by undermining and advancement	Extensive undermining may cause scrotal flap loss or wound edge necrosis
	Simple, safe and easy procedure	-
	Less recovery time	-
	Shorter hospital stays	-
	Acceptable cosmetic appearance	-
Split thickness skin grafting	contour well to irregular surface	Sensitive and prone to mechanical trauma
	Can be used in conjunction with local flap coverage	Scar contracture may be seen
	Simple, safe, easy and one stage procedure	Appearance in not completely natural since the new sac lacks redundant skin and testicles are not freely floating, instead testes remain in a low position due to loss of cremasteric function.
	Thin skin resembles normal scrotal skin, contour and shape	Pain, discomfort due to lack of mobility between grafted skin and testes also reported
	The testicular function is preserved because testis remains cool (testicle temperature low and may add spermatogenesis)	STSG cannot be performed if testes have been stripped of tunica vaginalis
	Decreased recovery time	-
	Shorter hospital stays	-
Medial thigh flap cover	technically simple	Sometimes mild oedema of lower limbs
	Advance easily to defects	Paresthesia of anterior surface of thigh
	Minimal disruption to donor sites	bulkiness of flap
	Single stage	impaired spermatogenesis as a result of difficulty in maintaining lower temperature in the testicle (35℃)
	Sensate flaps	-
	donor site closed primarily	-
	Functional expandability	-
	Achieve reasonable aesthetic result. Very vascular and safe to use, even in diabetic and vasculopathy patients	-
Anterolateral thigh flap cover	Sensate and large flaps	More difficult dissection with vascular anatomy variability
	The flap is out of zone of infection	cannot be used in poorly managed diabetic patients because prone to wound dehiscence and infection
	Have hair bearing skin	In obese patients the ALT flap would be thick and hence and unsuitable for coverage of scrotal defect
	Flap looks bulky in initial post operative period but in the long term the oedema subsides leaving a natural looking scrotum	
	Flap can be harvested as a musculocutaneous flap with part of vastus lateralis muscle that can be used to obliterate dead space	-
	flap can also be harvested as a neurocutaneous flap with lateral cutaneous nerve of thigh	-
Groin flap cover	simple technique	Multi-staged procedure
	Advance easily to cover defects	bulkiness of flap
	Minimal disruption to donor sites	impaired spermatogenesis as a result of difficulty in maintaining lower temperature in the testicle (35℃)
	donor site closed primarily	
	Functional expandability	
	Achieve reasonable aesthetic result	
	Flap is thin (specially on lateral side) as compared to other fascio-cutaneous flaps.	
	Testes maintains its retractile property	

## Discussion

Reconstruction of a scrotal defect poses a challenge after surgical debridement. The “replace like with like” principle has an important role in functional and aesthetic reconstruction of scrotal defects, which is difficult because of its unique texture, color and contour.

According to Chen et al. [10], when the scrotal defect area is less than half the scrotal surface area, scrotal advancement flap can be performed. Closure of scrotal defect with remnant scrotal tissue has an advantage of the testes being covered with native tissue so appearance is similar to the original scrotum. Since testes are covered with native tissue, spermatogenesis is affected minimally, in contrast to fascio-cutaneous flaps. There is no donor site morbidity. The procedure is safe and simple without the risk of any complications besides reducing the hospital stay and hence, cost as well. Chen et al. [11] reported successful scrotal defect reconstruction in 11 patients using the scrotal advancement flap. All patients had scrotal defects less than half the surface area of scrotum. Partial flap loss was seen in one patient and wound edge necrosis was seen in two patients in their study. We have also done scrotal defect resurfacing by scrotal advancement flap in 10 patients having scrotal defect less than 50% of scrotal surface area and all flaps survived well without any complications.

Balakrishnan was the first to use skin grafts to manage scrotal defects [12]. Split thickness skin grafting is a safe, cost-effective method of scrotal defect reconstruction that is technically easy to perform. The reconstructed scrotum is identical in color and shape to the original scrotum, besides, having thin skin which keeps the testis cooler than the normal body temperature. However, the newly constructed scrotum lacks the redundant skin. Also, testicles keep lying in a low position due to loss of cremasteric function [13]. Hesselfeldt-Nielsen and co-workers had reported three such cases of scrotal defect reconstruction with split thickness skin grafting with excellent results except for the lack of cremasteric suspensory function which was taken care of easily by using ordinary tight underpants [14]. Several authors have reported the use of split thickness skin grafting for scrotal reconstruction following Fournier’s gangrene. Parkash and Gajendran reported 43 cases of scrotal reconstruction following Fournier’s gangrene [15]. Altcheck and Hoffman [16] managed scrotal defect following Fournier’s gangrene using various reconstruction modalities such as split thickness skin grafting, myo-cutaneous flaps and placement of testis in subcutaneous thigh and abdominal wall pockets and concluded that the modality of choice for scrotal reconstruction should be split thickness skin grafting. We have also resurfaced scrotal defects in seven patients with split thickness skin grafting with acceptable aesthetic results.

A number of flaps have been reported in literature for scrotal reconstruction, however these flaps are bulky unlike normal scrotal skin. Besides, excessive fat content of thick fascio-cutaneous flaps can increase the temperature around testes resulting in inhibition of spermatogenesis. Due to complex techniques or multistage operations in some cases, the hospital stay is also prolonged adding to the monetary burden of patient. The only absolute indication for flap reconstruction is when vital structures such as the tunica, corpora and urethra are breached. Flaps, however, provide hair-bearing skin and more durable soft tissue than skin graft which is the chief advantage of using flaps for scrotal reconstruction. Flap coverage has reliable vascularity, durability, functional expendability. The fascio-cutaneous flap of inner thighs has excellent vascularization because of the presence of branches of the femoral artery (internal and circumflex pudendal), making the flap very reliable in diabetic and patients with ischemic disease [17]. Also, it can be done in short operative time to cover difficult defects. Hallock [18] reported the same flap for scrotal reconstruction following Fournier’s gangrene.

We have seen minor complications such as margin necrosis in two patients of medial thigh flap coverage however, both cases were managed conservatively and the flaps showed good sensation except for transient loss of sensation over anterior part of thigh which regained in the follow-up period. These advantages and disadvantages were comparable to that reported by Ferreira et al. [19] in their review of management of 43 patients of Fournier’s gangrene. Donor site complications such as wound dehiscence and infection of the donor site suture line were seen in two patients and one patient respectively. Direct closure was done for wound dehiscence and infection was managed by frequent dressing. These minor complications were comparable to that reported by Ferreira et al. [19].

Anterolateral thigh flap is another option for reconstruction of scrotal defect of Fournier’s gangrene. Anterolateral thigh flap is away from the zone of infection in cases of Fournier’s gangrene and bring healthy vascularized tissue into the defect. These flaps can cover defect up to 35×25 cm [20]. Anterolateral thigh flap can be harvested as fascio-cutaneous or musculocutaneous flaps with part of vastus lateralis or as neurocutaneous flap with anterior lateral cutaneous nerve of thigh. Anterolateral thigh flap has some limitations. In obese patients, anterolateral thigh flap would be thick so cannot be used for scrotal reconstruction. Chen et al. [11] reported reconstruction of three scrotal defects with pedicled anterolateral thigh flap cover with good outcome and survival. Spyropoulou et al. [21] used a pedicled ALT flap in nine patients with penoscrotal defect. According to the author, flap appeared bulky in initial postoperative period but oedema subsided during the course of time, leaving a more natural-looking scrotum. In, our study we used ALT flap in seven patients to reconstruct scrotal defect and all flaps survived well with good aesthetic outcome. However, the bulkiness of ALT flap remains its disadvantage. 

Groin flap technique is simple and flap advances easily to cover scrotal defects. Groin flap has functional expandability and achieves reasonable aesthetic results. In our cases results were also acceptable. The flap is thin and pliable as compared to other fascio-cutaneous flaps. This flap remains the choice when pliable tissue is required.

The choice of reconstruction technique of scrotal depends upon the site of defect, extent of scrotal involvement, age of patient, comorbidities and surgeon’s preference. Both delayed primary closure and STSG are safe, easy and single-stage procedures with shorter hospital stay. However, delayed primary closure is only possible for scrotal defect involving less than 50% of scrotal surface area and STSG cannot be done when tunica vaginalis is stripped off, although thin skin in STSG resembles a normal scrotum which preserves testicular function. Fascio-cutaneous flaps offer single staged, well-vascularized soft tissue coverage in scrotal defects. Medial thigh flaps can be used for coverage even in diabetic and vasculopathy patients. Anterolateral thigh flaps provide large, hair-bearing cutaneous pedicles that are out of zone of infection and can be harvested as musculocutaneous flaps to obliterate dead space in perineum whereas groin flaps offer thin expandable coverage.

Every modality used to cover scrotal defect has its own pros and cons, so every case needs an individual approach for scrotal reconstruction depending upon patient’s condition, patient’s requirement and surgeon’s expertise to achieve reasonable functional and aesthetic outcome without any major complication and donor site morbidity. 

The limitation of our study is a small sample size and lack of standardization, hence it is recommended to conduct randomized controlled trials to evaluate the different methods for soft tissue reconstruction of the scrotum.

## Conclusions

Each case of scrotal defect needs an individualized approach for tailoring the scrotal reconstruction depending upon patient’s age, general condition of patient, wound status, comorbidities, patient’s requirement and the advantages and disadvantages of the different management modalities will definitely help in decision-making for soft tissue reconstruction of the scrotum under optimal conditions.
